# The challenges of female chronic pelvic pain

**DOI:** 10.1590/1806-9282.706EDIT

**Published:** 2024-07-19

**Affiliations:** 1 Lim_58 laboratório de Ginecologia molecular e estrutural, Disciplina de Ginecologia, Departamento de Obstetrícia e Ginecologia, Hospital das Clínicas, Faculdade de Medicina, Universidade de São Paulo – São Paulo (SP), Brazil.

The topic of chronic pelvic pain is of paramount importance to the gynecologist because this entity compromises the quality of life of women with great prevalence. It is a reason for professional absenteeism and psychosocial disorders that affect the relationships of these women in all areas of their daily lives. The great challenge of the gynecologist in dealing with this entity is justified by the fact that the cause is often multifactorial and that commonly the disorders of the reproductive system can be associated with dysfunctions of the digestive system, urinary system, and musculoskeletal system.

Chronic pelvic pain is defined as persistent, acyclic pain perceived through pelvic structures lasting more than 6 months. It affects around 26% of the female population^1^, and its etiology may be associated with several diseases, and the concomitant presentation of pathologies is not uncommon, which occurs in up to 50% of cases^2^. Among the main physical causes of pelvic pain, endometriosis, adenomyosis, irritable bowel syndrome, chronic interstitial cystitis, myofascial pain syndrome, and pain associated with venous return insufficiency stand out. However, it is worth noting that among patients who seek medical care for chronic pelvic pain, about half will have some history of sexual, physical, or emotional abuse, with an important effect on pain modulation and perception. In addition, it is important to remember that there is an association between chronic pelvic pain and other non-pelvic disorders such as fibromyalgia and migraine^1^.

The International Society of Chronic Pelvic Pain has created a reference of symptoms and changes in physical examination to guide healthcare professionals in possible diagnoses present in women with chronic pelvic pain. After listening carefully to the patient's complaints, characterizing the type of pain, intensity, association with symptoms, triggering, and improvement factors is essential to aid in the diagnosis and consequently in the appropriate therapy^2^. Symptoms and their connection to the main differential diagnoses are highlighted: association of pain with the menstrual period (endometriosis and adenomyosis), cramp-like pain (inflammatory bowel disease), sensations of shock, burning, and heat (nerve compression), voiding pain and urgency (interstitial cystitis and urethral syndromes), postcoital pain and bleeding (cervical neoplasia), postmenopausal bleeding (endometrial neoplasia), weight loss (malignant and systemic diseases), and history of multiple surgical approaches (fibrosis and adhesion)^2,3^.

The Visual Analog Scale (VAS) is the most widely used for quantifying pain intensity and is extremely useful, including for post-treatment follow-up and improvement assessment. There are also symptoms considered "red flags" that should be prioritized over the investigation because they may be associated with the presence of neoplasia or serious systemic diseases, such as rectal bleeding, intestinal symptoms after the age of 50 years, pain that arises after menopause, pelvic mass, suicidal ideation, excessive weight loss, significant vaginal bleeding after the age of 40 years, and bleeding after sexual intercourse^3^.

It is essential to have discernment of the type of pain that the patient is referring to, if it is a pain related to the illness of some target organ, which in the vast majority of cases is what we think, known as nociceptive pain, if the origin of the pain is central (hypothalamic), known as nociplastic pain, or even if it is caused by irritation of some peripheral nerve, known as neuropathic pain^3,4^.

Patients with chronic pain for long periods can be victims of a process of neuroplasticity, thus changing their perception of the painful stimulus and amplifying the symptoms at the level of the central nervous system^4^. In addition, it is very common to come across cases of female pelvic pain, with mixed pain, of multiple origins.

For the treatment of these pains, surgery is often the solution, requiring the use of alternative therapies such as physiotherapy with myofascial release for patients with "trigger points" in the pelvic region, cognitive behavioral therapy, myorelaxant medications, tricyclic antidepressants, or selective serotonin reuptake inhibitors and GABA inhibitors (gabapentin and pregabalin)^4,5^. Great care should be taken with the improper prescription of opioids for the treatment of chronic pain due to the risk of addiction to drugs.

Among the various causes of chronic pelvic pain, we highlight the importance of three main non-gynecological causes: interstitial cystitis, myofascial pain, and irritable bowel syndrome.

## INTERSTITIAL CYSTITIS

Interstitial cystitis is defined as a chronic inflammation of the bladder and urinary tract whose main symptoms are suprapubic pain, dysuria, and urinary urgency. One of the standout features is pain relief on bladder emptying^6^. Symptoms of interstitial cystitis are commonly found in patients with chronic pelvic pain. In three observational studies of women with pelvic pain who sought treatment, around 38–84% had symptoms suggestive of interstitial cystitis^7-9^. The diagnosis is exclusionary, which sometimes delays the implementation of treatment.

Myofascial pain can arise from changes in the musculoskeletal system. Most patients with this etiology for pain have trigger points identified as areas of muscle band contracture that are very painful on palpation. It is believed that the appearance of trigger points may also be associated with pelvic misalignment, secondary to postural changes due to the effect of pain with its perpetuation. That is, they can be both a consequence and a cause of chronic pelvic pain^3^.

Irritable bowel syndrome is a disease that alters gastrointestinal functionality and is mostly characterized by abdominopelvic pain associated with changes in the frequency and formation of stool. It most often presents in relapses and remissions and is more common in patients with psychiatric comorbidities. Although there is no complete understanding of the pathophysiology, it is known that there is a hyperstimulus in the central nervous system with feedback of symptoms^10^.

Pelvic varicose veins also represent a concern in the workup in patients with pelvic pain, as there is a direct relationship between pelvic pain and venous compression syndromes that may promote pelvic congestion. This presents as a hard type of pain with worsening at the end of the day and associated lower limb edema in most cases^11^.

Compressive syndromes may be related to the presence of arteriovenous malformations that hinder the venous return of the female reproductive system, such as Cockett's syndrome (left common iliac vein is compressed between the right common iliac artery and spine) and Nutcracker syndrome (left renal vein and superior mesenteric artery and aorta)^11^.

For the diagnosis, the use of magnetic resonance angiography of the abdomen and pelvis with contrast is necessary, and the treatment requires expertise in the application of stents by interventional radiology^11^.

Adenomyosis is also a benign gynecological disease of high prevalence and may or may not be associated with endometriosis, which is considered an important cause of chronic pelvic pain with symptoms that vary according to the degree of involvement^1,3^. It evolves from cyclical pain such as dysmenorrhea, which may or may not exacerbate menstrual blood flow, promote dyspareunia and infertility, and also be considered a cause of chronic pelvic pain^3^.

Hormonal treatment with progestogens has good results, but it is restricted to women without reproductive desire or with constituted offspring. In cases of pain refractory to medical treatment and without reproductive desire, total hysterectomy can be an excellent alternative, but it is important to make it clear that in cases where there is an association with deep endometriosis, if it is also not removed during surgery, there may be the persistence of all pain symptoms^2,12^.

According to epidemiological studies, endometriosis represents around 50% of the causes of chronic female pelvic pain and is therefore considered the main cause of pelvic pain^12^. In the last 20 years, we have been following the evolution of non-invasive diagnostic imaging through ultrasound with bowel preparation and magnetic resonance imaging of cases of deep peritoneal endometriosis^13^.

Despite the concept that there is no direct relationship between the degree of endometriosis involvement and the level of pain presented by the patient, information on the presence of the disease through imaging tests is considered a positive predictor that justifies the cause of pain. In these cases, considering the absence of some conditions such as reproductive desire, signs of intestinal or ureteral subocclusion, presence of large-volume endometriomas, appendix, and ileocecal endometriosis, clinical treatment may be chosen instead of immediate surgical treatment^12,13^, and surgical treatment is restricted to cases of refractoriness to clinical treatment and to the absolute indications previously mentioned.

Surgical treatment, on the other hand, is still a great challenge because deep endometriosis surgery is highly complex due to the distortion of the anatomy, which can lead to infiltration of the retroperitoneum toward vital structures such as the ureter, large vessels, and autonomic and somatic pelvic innervation, in addition to intestinal involvement (rectum, sigmoid, ileum, cecum, and appendix), bladder, and diaphragmatic^12-14^. Therefore, there is a need for a multidisciplinary team for complete resection of the disease. For this reason, there is a very large percentage of patients who undergo suboptimal surgeries with the persistence of the disease and therefore the persistence of symptoms.

The use of hormonal therapy in the postoperative period has been pointed out as a fundamental tool in the attempt to prevent the secondary recurrence of lesions and symptoms^14^.

Another less frequent cause, but which we cannot rule out, is pelvic-perineal pain related to damage to the pudendal nerve that can be caused by accidents, bruising, or more often by stretching of the nerve associated with childbirth, pelvic organ prolapse, sports such as cycling, or even patients who remain seated for long periods^15^. The pain pattern is often associated with shock, paresthesia, and burning sensations and can be relieved with changes in positioning that tend to decompress the nerve root^3^. Another therapeutic alternative is imaging-guided infiltration of the Alcock canal with local anesthetics by imaging^15^.

Female chronic pelvic pain is indeed a huge challenge for the gynecologist. The latter should be aware of several possible etiologies, both gynecological and non-gynecological, and may require expertise in diagnosis that goes beyond organic pain of organs located in the pelvis and multidisciplinary team effort for the best treatment in the search for long-lasting results.
